# Farmers’ knowledge on cultivation, utilization and conservation practices of barley (*Hordeum vulgare* L.) in three selected districts in Ethiopia

**DOI:** 10.1186/s13002-022-00556-2

**Published:** 2022-09-04

**Authors:** Eyasu Wada, Abas Abdulahi, Tekuamech Fikadu Tehelku, Meseret Ergando, Hewan Demissie Degu

**Affiliations:** 1 Department of Biology, College of Natural and Computational Sciences, Wolaita Sodo University, Wolaita Sodo, Ethiopia; 2 Depatrment of Ecotourism and Biodiversity Conservation, College of Agriculture, Bedelle Campus, Mettu University, Bedelle, Ethiopia; 3 Department of Horticulture, College of Agriculture, Wolaita Sodo University, Wolaita Sodo, Ethiopia; 4 Plant Biotechnology, School of Plant and Horticulture Sciences, College of Agriculture, Hawassa University, Hawassa, Ethiopia

**Keywords:** Barley, Commercial cultivars, Local varieties, Indigenous knowledge

## Abstract

**Background:**

Farmers’ knowledge has a role in maintaining barley (*Hordeum vulgare* L.) genetic resource, which plays an important role in food security, and provides socio-cultural value to the Ethiopian farmers. However, farmers’ knowledge has been ignored in the decision-making process in Misha, Gumer, and Hetosa districts, Ethiopia.

**Methods:**

In this study, a semi-structured interview guide was used to carry out comprehensive house-to-house interviews with 357 purposively selected farmers to document their knowledge of barley cultivation, utilization and conservation practices.

**Results:**

The majority of farmers (57.1%) grow barley on 0.5–0.75 hectares. Farmers identified and described 68 barley varieties with various local names, which were given to barley based on different characteristics such as plant height, spikelet length, row type, seed size and color, yield, place of origin, and use-values. Farmers are familiar with the nature, characteristics, end-uses, and preparation of different well-appreciated local meals and drinks. Farmers noticed that the number of barley local varieties has been decreasing in recent years. Introduction of improved varieties was perceived by all farmers as the main cause for the decrease in the number of barley local varieties in their localities. Another factor for the reduction in local barley varieties, according to 24.2% of farmers, was soil fertility degradation. Most of the farmers (65.7%) use their own barley seeds, which they select and save for the next growing season for specific attributes. They have their own indigenous knowledge that they have acquired through experience by growing, selecting, and conserving barley for the last 20–30 years or more.

**Conclusion:**

The majority of farmers gave attention to commercial cultivars due to their better market value. Thus, the introduction of improved cultivars has imposed on local varieties. The indigenous knowledge that the famers acquired through experience could be considered an advantage for the conservation of barley genetic resources by using farmers’ participatory approach to widen cultivation and to improve barley local varieties for future use.

## Introduction

Barley (*Hordeum vulgare* L.) is a member of the grass family, Poaceae. All cultivated barleys are self-fertilizing, diploid annuals (2*n* = 14), either two-or six-rowed, but some six-rowed cultivars appear to have only four rows of kernels. Thus, reference is sometimes made to four-rowed barleys, although these are really six-rowed barleys. The spike, or head of barley, consists of a series of spikelets that are attached at nodes to alternating sides of the rachis. Each spikelet contains a floret [1, 2]. It is cultivated globally and grows successfully in diverse eco-geographical regions in a wide range of environments with an altitude range of 1500 to 3500 m above sea level (m.a.s.l). It tolerates soil salinity, drought, and frost to a considerable level [3]. It is the fourth most important cereal crop in the world after wheat, maize, and rice [4]. In addition to South Africa, Kenya, Egypt, Algeria, and Ethiopia are the top five barley-producing African countries [5].

Ethiopia is well known for its diverse native barley types and is recognized as a center of diversity for barley [6, 7], which is evenly distributed over the barley-growing areas of the country [6]. Barley producers of the country have given the name ‘*Gebis ye ehil nigus*’, which means barley is the king of all crops due to its suitability for preparing different kinds of known Ethiopian traditional dishes [8].

Farmers' knowledge of their varieties contributes to a better understanding of the genetic basis of environmental adaptation and the efficient use of genetic resources [9]. The skills with which farmers recognize and manage a given amount of diversity have important evolutionary consequences for a crop species [10]. Thus, recognizing the farmers’ varieties and traditional systems of characterization, cultivation, utilization, and conservation is important to conserve genetic resources, which were preserved from generation to generation [11]. An understanding of farmers’ knowledge is essential for planning research and development activities and in situ conservation strategies [12]. Understanding the sociodemographic factors that influence farmers’ decision-making is also crucial for the future improvement of a crop species [13].

In Ethiopia, barley cultivation is mostly of landraces that are chosen by farmers for suitable end-use or for adaptation to specific farming systems [14]. The use of barley and its value in the socio-cultural context to maximize on-farm productivity play a critical role for the maintenance of various barley varieties to ensure farmers’ household food security [15], which are the potential sources of adaptation to harsh agroecosystems [16]. Currently, barley genetic resources are exposed to the high rate of genetic erosion and are seriously endangered in the country [17].

From a genetic resource utilization and conservation point of view, there is a potential to exploit the genetic differences by making use of farmers’ knowledge, as the names that farmers give to varieties is the unit that they manage and select over time [18, 19]. In this regard, farmers’ knowledge and on-farm diversity of barley was assessed in Bale and North Shewa [20], Tigray region [18], Welmera and Ejere districts [21], highlands of North Gondar [22], northwestern parts [6] and Bale high lands of Ethiopia [23].

The Misha district of Hadiya zone, Gumer district of Gurage zone, and Hetosa district of the Arsi zone are among the major barley producing areas of Ethiopia. Farmers in these areas have a wealth of knowledge about seed practices that have been passed down through generations. Barley is one of the stable foods for farmers in these districts, and its production and food consumption take the lion's share of food security. However, farmers' indigenous knowledge on the cultivation, utilization, and conservation practices of barley in these districts has not been studied and documented. This study was, therefore, initiated to document barley varieties (local and improved cultivars) based on farmers’ indigenous knowledge and to determine the cultivation, utilization, selection, and conservation practices undertaken by farmers on the barley grown in the districts. This study could play an important role in enhancing barely varieties, which are being used as a solution for protecting food security in resource-poor farming systems, meeting future food needs and providing social benefits for a rapidly growing population.

## Materials and methods

### Description of the study area

**Fig. 1:**
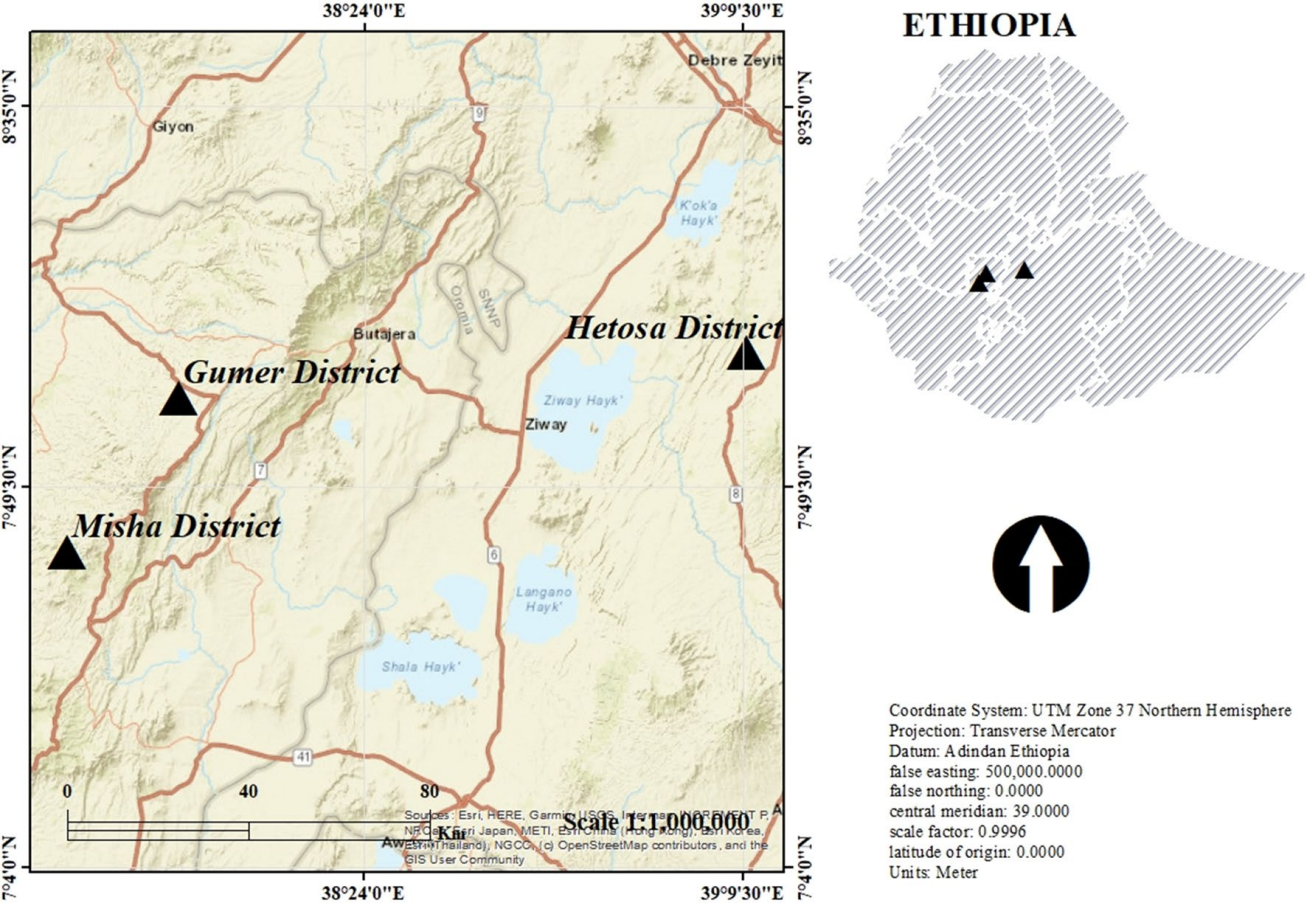
Location of study districts

The study was conducted in three selected districts (Misha district of Hadiya zone; Gumer district of Gurage zone; and Hetosa district of Arsi zone) in Ethiopia (Fig. 1). The districts were selected purposively based on their record in barley cultivation. The Misha district of Hadiya zone is characterized by being sloppy and flat with a humid tropical climate. The altitude ranges from 1820 to 2950 m.a.s.l with temperature ranges from 18 to 25 °C and rainfall ranges from 1000 to 1500 mm. The district fully experiences livestock and crop production. Rice, potato, cabbage, wheat, beans, carrot, tomato, beetroot, apple, and peach are all major food crops. The average temperature and rain fall of the Gumer district range from 12.6 to 22.5 °C and 1001 to 1400 mm, respectively. The major food crops grown in the district are enset, potato, barley, wheat, peas, beans, radish, carrot, and cabbage. The plains of Hetosa in the Arsi zone are characterized by low land features. The altitude of the district ranges from 2332 to 3065 m.a.s.l. The mean annual rainfall ranges from 800 to 1300 mm, and the average annual temperature is 10.25 °C. The major annual crops grown are wheat, barley, teff, maize, horse beans, haricot beans, field peas, linseeds, and rapeseed.

### Conceptual approach

Ethiopian farmers have detailed knowledge about different crops that they grow and identify agronomic attributes such as resistance to pests and diseases, drought tolerance, suitability for cultivation, and keeping quality [24–27]. They have a rich cultural heritage and traditional knowledge passed down through generations. They use seeds that they grow on their private farm, select, manage, and conserve for years. They also obtain seeds for cultivation from markets and neighbors [18]. The Alliance for Food Sovereignty in Africa reported that small-scale farmers are real seed producers as they prefer seeds that are stored locally, require no cash outlay, and can be relied upon to produce nutritional value [12]. The decline in the number of local varieties of barley is explained by several interrelated factors [23]. The socio-cultural background of the farmers was taken into consideration as an input to identify the farmers' knowledge about the cultivation, selection, and conservation of barley varieties. The meanings of the local names given to the barley cultivars were also taken into consideration when conducting the current study. A survey was conducted from July to December 2021 to collect the farmers’ knowledge of barley. In the course of this manuscript, we have used the terms “local variety” to refer to barley landraces identified by farmers; the term “improved barley variety” for commercial barley cultivars, and the term “barley variety” was used to refer to both landraces and commercial barley cultivars.

### Sampling strategy

The districts were selected purposively based on their record in barley cultivation. Kebeles (the smallest administrative units) were also purposely selected in terms of barley production potential. The selection was made after a preliminary survey and discussion with the agricultural experts of each district and the plant experts of the selected kebeles. A total of 357 (114 to 123 farmers) who have experience in growing barley were purposefully selected with the assistance of the leaders and the plant experts of the farmers’ associations of each kebele.

### Data collection

An intensive house-to-house interview was conducted in the respondent’s native languages of the respective ethnic groups (Hadiya, Gurage, or Oromo) using a semi-structured interview guide. Before collecting farmers’ perception, they were informed about the purpose of the research and its benefits, clearly underlining the fact that the results will be used for academic purposes and that no commercial interest will be attached to it. A verbal agreement was obtained from the authorities of local communities prior to administering the interview. When farmers assertively stated that this research is useful and agreed to provide the required information, they were asked for local names, preferred traits, row types, seed color, local foods and drinks prepared from barley, the number of barley varieties that they used to cultivate or that they cultivate currently, sources of seeds used for cultivation, utilization, selection, and conservation practices. Field observations were made on barley fields, farming systems, and conservation practices.

### Data analysis

Data were coded in Microsoft Excel and analyzed using the Statistical Package for Social Scientists (SPSS) version 23 [28]. The association between the age of the farmers and barley cultivation experience; land size owned by farmers and the number of barley varieties; and the amount of barley produced at a household level for home consumption and for sale was tested by an independent sample t test. There was a person correlation between the farmers’ age and their barley cultivation experience; the farmers’ age and the number of barley that they cultivate; the farmers' educational level and their barley cultivation experience; and the farmers' educational level and the number of barley that they cultivate. The gender and age distribution of the respondents were tested using a Chi-square goodness-of-fit-test in Minitab 2013 [29]. Local barley varieties were listed using the local names and their meanings. Content analyses were conducted to assess the variation of barley by local names, row type, seed color, and farmers' preferred and non-preferred traits. The data was presented as frequencies and percentages of farmers sampled.

## Results

### Sociodemographic characteristics of the farmers

**Fig. 2:**
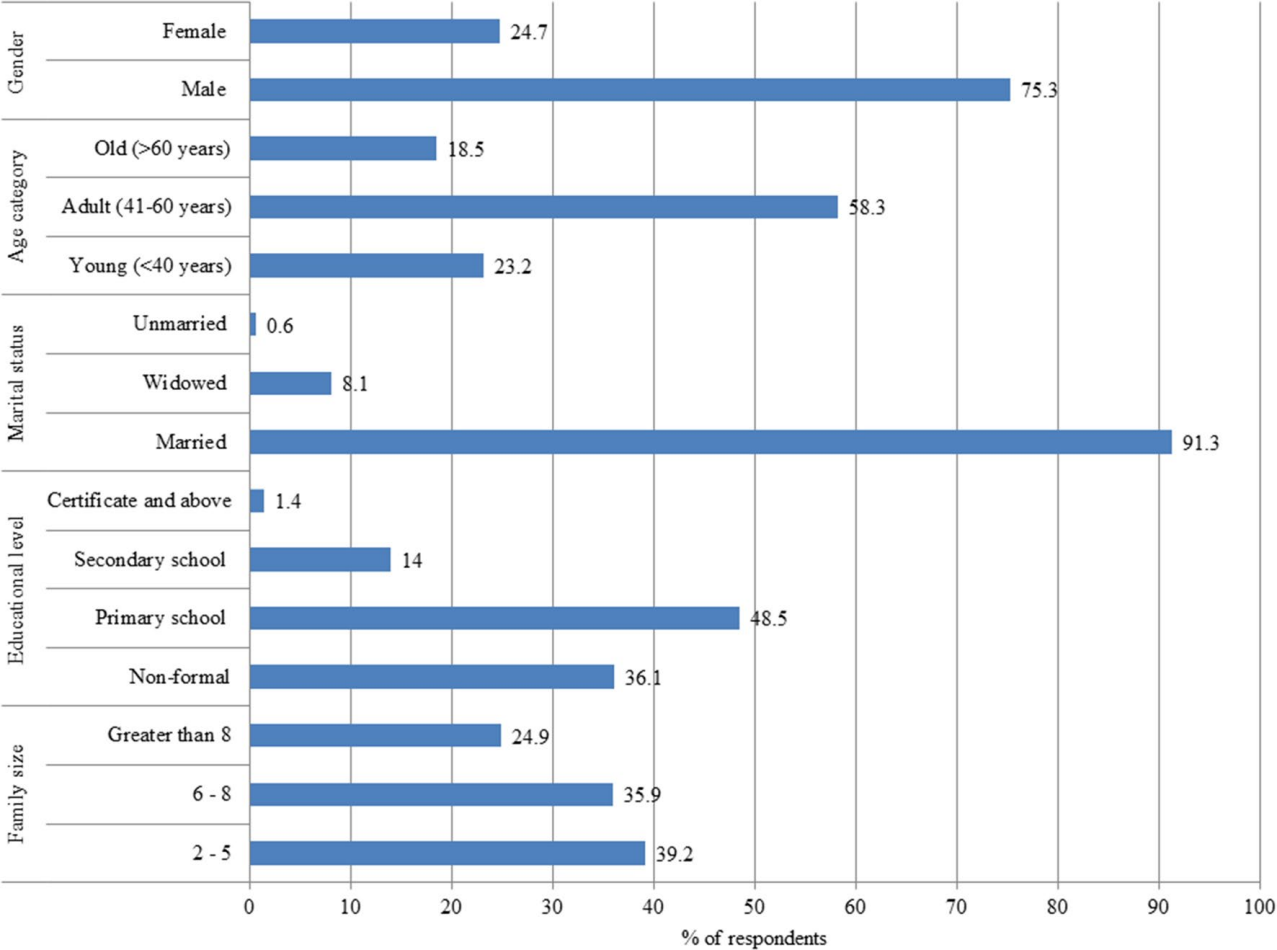
Sociodemographic characteristics of the respondents

The sociodemographic characteristics of the interviewed farmers are presented in Fig. 2. Accordingly, 75.3% of them were male, while a significantly lower proportion of female farmers (24.7%) were interviewed (*x*^2^ = 91.77, *df* = 1, *p* 0.001). Regarding age groups, the number of interviewed farmers who were between 41 and 60 years old (58%) was significantly higher than those who were 40 years old (23.2%) and > 60 years old (18.5%) (*x*^2^ = 28.38, *df* = 2, *p* 0.001). Farmers' ages and barley cultivation experience are significantly and positively Pearson correlated (*r* = 0.90, *P* < 0.001), but negatively correlated with the number of barley varieties cultivated (*r* = − 0.181, *P* < 0.001). Most of the interviewed farmers (91.3%) were married, followed by widowed (8.1%). The unmarried farmers were the least represented (0.6%). The majority of farmers (48.5%) completed primary education along with agriculture work, while 36.1% did not complete formal education, and only 1.4% had a certificate or above award for formal education. More educated farmers have less experience in the cultivation of barley landraces (*r* = − 0.375, *p* < 0.001). Educational level was also negatively correlated (*r* = − 0.079) with the number of barely varieties that the farmers cultivate, although the correlation is not significant (*p* = 0.134). The majority of farmers (39.2%) have two to five children.

### Barley growing experience of farmers’ and land size

**Fig. 3:**
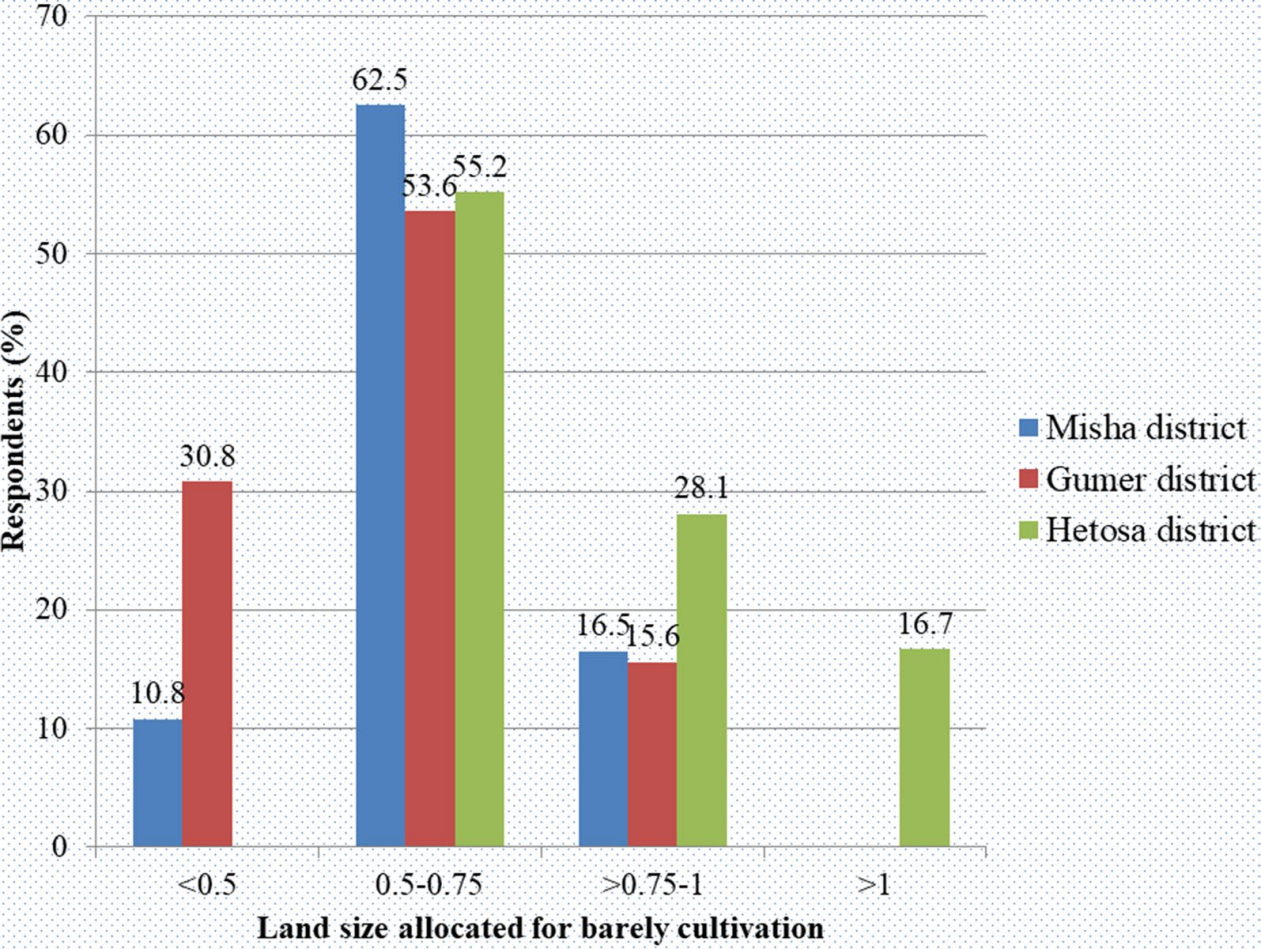
Land size allocated for barley cultivation

The information generated during this study was obtained from farmers who had been cultivating barley for a period of 4–55 years. According to farmer responses, the majority (55.6%) have been using local barley varieties for more than 20 years. In the Hetosa district, all farmers grow barley on ≥ 0.5 hectares of land (Fig. 3), and the greatest number of farmers (59.7%) reported having been growing barley for the last 31 years or more. The greatest number of farmers in Misha has the shortest period of barley cultivation experience (Table 1). The majority of the farmers (57.1%) grow barley on 0.5–0.75 hectares, followed by those who grow on > 0.75–1 hectares. Among the three study districts, farmers who grow barley on > 1 hectare were encountered only in the Hetosa district of the Arsi zone.

**Table 1:** Barley cultivation experience of farmers

Years	Number of respondents*			
	Misha district	Gumer district	Hetosa district	Total
< 10	22 (18.3)	25 (20.3)	4 (3.5)	50 (14)
11–20	36 (30)	62 (50.4)	12 (10.5)	108 (30.3)
21–30	42 (35)	35 (28.5)	30 (26.3)	107 (29.9)
31–40	20 (16.7)	1 (0.81)	36 (31.6)	58 (16.4)
41–55	–	–	32 (28.1)	34 (9.4)

*numbers in parentheses are the percentage of respondents

### Local names, their meanings and farmers’ preferred features of barley

The barley varieties cited by farmers, local names of barley varieties along with their meanings, seed color, number of rows, and summary of farmers’ preferred and non-preferred traits are presented in Table 2. A total of 68 barley varieties (landraces and improved) with distinct local names were reported by the interviewed farmers. Of these, 22 were recorded from Misha district, 15 from Gumer district and 31 barley varieties were recorded from Hetosa district. Farmers use different characteristic features of barley, such as length of spikelet, dehulling, digestibility, row type, seed size, seed color, yield, kernel color, plant height, and place of origin, use values, and the person who introduced the barley to the locality for the first time to assign local names.

**Table 2:** Local names of barley varieties cited by farmers, along with their meanings, seed color, number of rows, and summary of farmers preferred and non-preferred traits

District/Local language	Local names of barley varieties	#Record	# rows	Seed color	Meaning of local names	Farmers’ preferred traits	Non-preferred traits of barley
Misha district in Hadiya Zone/Hadiya	*1. Awodo*	106	2	White	Means poor people milk to indicate that it is used as a milk substitute its white kernel color	High nutritional value, has sweet taste	Late mature, low yield, difficult to dehull
	*2. Du’uyya*	98	6	Black	Named to indicate rounded short spike with black seed	Early mature, high yield, hull less	Does not have good taste
	*3. Nazena*	78	2	Black	Named to indicate good flavor and tasty	Provides strength and energy, good flavor, tasty, hull less and has medicinal value (used to treat animals to relieve from tapeworm)	Late mature, low yield
	*4. Gibrin-So’o Yebira-Gebis* (improved)*	69	6	White	Means that the farmers obtained it from agricultural office	High yield and easy to dehull	–
	*5. Anishicho*	17	2	Black	Meaning harmless to indicate awn less spikelet	Early mature and has medicinal value (to release the remains after birth)	–
	*6. Heemach-Wongara*	14	2	Light- gray	Named to indicate no need of labor to dehull with black color and large seed	Early mature, high yield, hull-less, sweet taste	–
	*7. Qadal-Wongara*	14	2	Light- yellow	Named to indicate easily for dehull with white color and large seed	Hull-less, sweet taste	–
	*8. Gorxena*	8	2	Brown	Named to indicate it is not easily digestible	Hull-less, provides strength when eaten	Late mature, low yield
	*9. Caanfo’o*	3	Irregular	Gray	Named to indicate irregular of rows	–	Low yield
	*10. Calqo’o*	3	6	Black	Named to indicate curved spike with large seed size	Early mature, high yield	–
	*11. Heemach-Xa’maja*	2	6	Black	Named to indicate its black seed color that make discomfort in stomach	Hull-less	–
	*12. Gardaama*	2	6	Light- yellow	Meaning cave honey to indicate medicinal value and too thick during preparation	Has medicinal value (maintain the broken bone)	Hull and difficult to dehull
	*13. Giraaryya*	1	6	Black	Meaning *Acacia* like named to indicate hard awn	High yield, hull less	–
	*14. Sheme'e*	1	2	Black	Named to indicate thin seed size	–	Low yield
	*15. Gooficho*	1	6	Black	To indicate origin from Gofa zone	Early mature, high yield	Less sweet
	*16. Olika*	1	6	Black	To refer long spike and large seed size	High yield	–
	*17. Hagala*	1	6	Gray	Named to indicate undifferentiated or multi-color seed	–	Low yield, less sweet
	*18. Yebira-Gebis* (improved)*	1	6	Light- yellow	Name to refer as it is used for beer making	High yield	Low energy value
	*19. Kashar-Xa'maja*	1	6	Light- yellow	Named to indicate its light yellow seed that discomfort stomach	Hull-less	–
	*20. Qadal-Du'uyya*	1	6	White	Named to indicate rounded short spike with white seed	–	Low yield
	*21. Timibra*	1	6	White	Means spike covered by additional husk	Sweet, hull less	–
	*22. Mirt-Tenfis*	1	6	White	Named to refer high amount of yield	High yield	–
Gumer district in Gurage zone/Gurage	*23. Jimua-Tikur*	87	6	Black	Name to refer short spike with black seed color	Early mature, high yield drought tolerant, disease resistant	Low market demand
	*24. Shege* (improved)**	81	6	White	Name to refer long spike	Long spike, high yield, high market demand	Less food quality
	*25. Nech-Senef*	62	2	Yellow	Named to mean ‘lazy white barley’	Long kernel, high market value, easy to dehull	Require fertile soil, low yield, susceptible to disease
	*26. Awodo*	56	2	White	Named to refer bright white kernel color	White seed color, best food quality	Intermediate yield
	*27. Chifeye-Awedo*	40	2	White	Named to refer kernel is covered by an additional husks	White seed color, best food quality, medicinal	Intermediate yield
	*28. Tikur-Senef*	17	2	Black	Named to mean ‘ lazy black barley’	Long kernel, easy to dehull	Low yield, low market demand, susceptible to disease
	*29. Yebira-Gebis*	12	2	White	Name to refer as it is used for beer making	High yield, high market demand	Seed high cost
	*30. Chelko-Tikur*	12	6	Black	Named to refer to long spike with black seed color	Early mature, drought tolerant, disease resistant, high yield, long spike	Shattering problem, low market demand
	*31. Jimua-Nech*	2	6	White	Named to refer short spike with white seed color	Early mature, high yield	Low market demand due to low food quality
	*32. Nech-Temezhe*	1	2	White	Named to refer white hulled kernel	Early mature, easy for dehull	Low yield, low food quality
	*33. Tikur-Temezhe*	1	6	Black	Named to refer black hulled kernel	Early mature, easy for dehull	Low yield, less food quality
	*34. Keleme*	1	6	White	Named to refer small sized kernel	Sweet taste, easy for dehull	Disease susceptible, low market demand
	*35. Shemeya*	1	2	Purplish red	Named to refer purplish red kernel color	Medicinal value, disease resistant, drought tolerant	Late mature, low yield, low demand
	*36. Wesabo*	1	6	Gary	Named to indicated variegated color (black and white)	Drought tolerant, high yield, disease resistant	Low market demand due to its gray seed color
	*37. Shehabdo/Dirgim efis*	1	6	White	Named to refer many yield	High yield, long spike	Low market demand due to low food quality
Hetosa district in Arsi zone/Oromo	*38. Walia*	25	6	White/Back	Named to reflect the endemicity as *Walia Ibex*	Short plant height and short spikes	Susceptible to drought and cold
	*39. Kabe*	19	6	White	Named after the person ‘Kabe’	Short spike and tolerant to lodging	Small sized low quality seeds
	*40. Wolkari*	10	2	White	To refers it is an improved variety	Short spikes, short plant height, tolerant to lodging	Susceptible to drought and cold
	*41. Eboni*	9	2	White	Improved and high yield barley	Short plant height, short spikes	Low seed quality, drought susceptible to
	*42. Miskali*	6	2	White	Improved and high yield	Long plant height, long spikes, good flour	Susceptible to drought and cold
	*43. Tesfaye*	5	6	Purple	Named after the person ‘Tesfaye’	Long spike, drought tolerant	Low seed quality, low market demand
	*44. Garbu-Guracha*	3	6	Black	Named to show its being black color	Long spikes, cold tolerant	Low market demand due to its seed color
	*45. Aruso-Guracha-Rogmale*	3	Irregular	Black	specify its origin ‘Arsi’ with its black color and irregular row	Long spikes, tolerant to drought, good flour quality	Low market demand due to its seed shape
	*46. Aruso-Magala-Rogmale*	3	Irregular	Brown	Named to specify its origin ‘Arsi’ with its purple color and Irregular row	Long spike, resistant to drought and cold, good flour quality	Low yield, low market demand due to its seed shape
	*47. Kate-Adi*	2	2	White	Named to show its seed looks line wheat seed	Short plant height, tolerant to lodging	Low yield
	*48. Aruso-Adi-Bate*	2	2	White	Secify its origin ‘Arsi’, whitish color and 2 rows	Tolerant to stress, good flour quality	Low yield
	*49. Abola*	1	2	White	–	Long plant height, large spike, large seed size,	Low food quality
	*50. Achachi-Bera*	1	2	White	Given to reveal its stunted plant height and its end-use	Short plant height, good flour quality	Susceptible to drought and cold stress, low quality
	*51. Akalas*	1	2	White	Given to show it withstands lodging	Short plant height, drought tolerant	Low seed quality and low yield
	*52. Abdo*	1	6	White	Named after the person ‘Abdo’	Small seed size, short plant height,	Low yield, needs fertile soils
	*53. Aruso-Guracha-Bate*	1	6	White	specify its origin ‘Arsi’ with its black color and two row	Small seed size, short plant height,	Low yield, needs fertile soils
	*54. Aruso-Magala-Bate*	1	2	Brown	Refers its origin ‘Arsi’ with its purple color, two row	Long spike, resistant to drought and cold, good flour quality	Low yield and low market demand
	*55. Aruso-Guracha- Diribi*	1	6	Black	Refers its origin ‘Arsi’ with its black color and six row	Long spikes, tolerant to drought, good flour quality	Low market demand due to its seed color
	*56. Feresgama*	1	6	Brown	Named to show its short plant and spikelet length	Short plant height, tolerant to lodging	Low market demand due to its small sized seed
	*57. Garbu-Guracha-Rogmale*	1	irregular	Black	Named to show its black color and its irregular row	Have long spikes, resist to cold stress	Low market demand due to its black seed color
	*58. Garbu-Guracha-Bate*	1	2	Black	Named to show its black color and its two row	Have long spikes, resist to cold stress	Low market demand due to its black seed color
	*59. Jilcha-Adi-Rogmale*	1	irregular	White	Given to reveal its difficulty digestion and its white color	Large spike, tolerant to lodging due to its short height	Low food quality and market demand, difficult for digestion
	*60. Jilcha-Magala-Bate*	1	2	Brown	Given to reveal its difficulty digestion and its brown color	Large spike, tolerant to lodging due to its short height	Low food quality and market demand, difficult for digestion
	*61. Jilcha-Guracha-Bate*	1	2	Black	Given to reveal its difficulty digestion and its black color	large spike, seed size and plant height, tolerant to lodging	Low food quality and market demand, difficulty for digestion
	*62. Karamba*	1	irregular	Brown	–	Large spike, good flour quality	Low yield
	*63. Luka’a-Guacha* (*Tikur-Senef*)	1	6	Black	Named to mean it is easy to dehull and its black seed color	Long spikes	Low yield
	*64. Luka’a-Adi* (*Nechi-Senef*)	1	6	White	Named to mean it is easy to dehull and its whitish color	Long spikes, mainly used for roasted barley	Low yield
	*65. Damoye*	1	2	Purple	Named to reflect its being spiky	Many spikes	Low yield, prefers fertile soil
	*66. Shamame*	1	2	Brown	Named to show its hard seeds	Drought, cold and lodging tolerate, has good tillers	requires fertile soil, low yield, low seed quality, low market demand
	*67. Samareta*	1	irregular	White	Named to reflect its attractive whitish-purple seed color	Large spike, seed size, and plant height, susceptible to lodgings	Low seed quality
	*68. Shege*	1	6	White	Used to refers it is an improved variety	High yield, high market demand	Less seed quality

^*^Yebira Gebis is cultivar introduced into Ethiopia through Holata Agricultural Research Center/EIAR

^**^Shege- a pure line selection from Ethiopian Biodiversity Institutes with the passport data ‘1622–05’released in 1995 by Holata Agricultural Research Center/EIAR, Ethiopia

Farmers' preferred barley has characteristics such as seed and food quality, seed color, flour quality, flavor, taste, hull less, early maturity, high yield, high market demand, ease of dehulling, large spikelet, disease resistance, and drought and lodging tolerance, while low yield, late maturity, requirement of fertile soil, susceptibility to disease and drought, shattering problems, and low market demand are among the traits that were reported by farmers as non-preferred characteristics of barley.

### Barley varieties by seed color and row type

White seed-colored barley varieties were the most widely distributed, encountered at 41.1% of farmers’ fields, followed by black seed-colored barley varieties, which were encountered at 29.4% of farmers’ fields. Regarding the row type, six row type barley varieties, which were identified by most farmers as high yielding, were the most widely distributed barley varieties, being recorded in 47.1% of farmers’ fields. Irregular row type barley varieties were the least recorded (10.3%) (Table 3).

**Table 3:** Distribution of barley varieties by seed color and row type

Seed color	Farmers		Row type	Farmers	
	Number	%		Number	%
Black	20	29.4	6	32	47.1
Brown	7	10.3	2	29	42.6
Gray	3	4.4	Irregular	7	10.3
Light-gray	1	1.5			
Light-yellow	5	7.4			
Purple	2	2.9			
Purplish red	1	1.5			
White	28	41.1			
Yellow	1	1.5			

### Status of the number of barley varieties used for cultivation

In the last 20–30 years, 124 farmers (34.8%) have grown five or more barley varieties. Currently, however, 253 (70.4%) farmers grow a maximum of 3 barley varieties (Table 4). An independent t test showed that the mean number of barley varieties (4.77) cultivated by farmers at household level before 2–3 decades was significantly greater than the number of barley varieties currently cultivated on their farm (2.66) (*P* < 0.001). The number of barley local varieties cultivated at household level has been decreasing over recent years although the frequency varies from district to district. The introduction of improved barley cultivars was the main reason for the decrease in the number of barley local varieties as perceived by all of the interviewed farmers. Soil fertility loss, land size decrease, climate change, low yield of some barley local varieties and low market demand were other reasons, which were reported by 40.1%, 3.4%, 59.1% and 24.1% of farmers, respectively. Fluctuation of rainfall, which affects the date of sowing, maturation, and harvesting, was also reported by some farmers as a reason for the decrease in the number of barley local varieties.

**Table 4:** Number of barley cultivated per household based on farmer responses (Number of respondents and percentage in bracket)

District (# respondents)	Time	Number of barley varieties						
		1	2	3	4	5	6	> 6
Misha (120)	20–30 years ago	0 (0)	0 (0)	0 (0)	0 (0)	6 (5)	14 (11.7)	100 (83.3)
	Current	0 (0)	2 (1.7)	54 (45)	41 (34.5)	20 (16.7)	3 (2.5)	0 (0)
Gumer (123)	20–30 years ago	0 (0)	3 (2.4)	65 (52.9)	53 (43.1)	2 (1.6)	0 (0)	0 (0)
	Current	0 (0)	38 (30.9)	46 (37.4)	34 (27.6)	5 (4.1)	0 (0)	0 (0)
Hetosa (114)	20–30 years ago	73 (64)	24 (21.1)	13 (11.4)	2 (1.8)	2 (1.8)	0 (0)	0
	Current	73 (64)	28 (24.6)	12 (10.5)	1 (0.9)	0 (0)	0 (0)	0 (0)
Three districts (357)	20–30 years ago	73 (20.4)	27 (7.6)	78 (21.8)	55 (15.4)	10 (2.8)	14 (4.0)	100 (28)
	Current	73 (20.4)	68 (19.0)	112 (31.4)	76 (21.3)	25 (7.0)	3 (0.84)	0 (0)

### Purposes of barley cultivation and production at household level

Primary purposes for the cultivation of barley and the mean amount of barley produced at household level (kg) in the study districts are presented in Table 5. The study indicated that most farmers cultivate barley for both home consumption and sale. In the Misha district, the mean amount of barley produced for home consumption (329 kg) was not significantly different from the mean amount of barley varieties produced for sale (361 kg) (*P* = 0.167). In the Gumer district, however, the mean amount of barley varieties produced at the household level for consumption (816 kg) was significantly lower than the amount produced for sale (1451 kg) (*P* < 0.001). Similarly, in Hetosa district, the mean amount of barley varieties produced at household level for home consumption (2057 kg) was significantly lower than the amount produced for sale (2450 kg) (*P* < 0.05). In general, in the study districts, the amount of barley produced at household level for sale (1420 kg) was significantly greater than the mean amount of barley produced at household level for home consumption (1067 kg) (*P* < 0.001) (Table 5).

**Table 5:** Purpose of barley cultivation and the amount of barley production

District	# respondents	Purpose of barley cultivation (%)			Mean amount of barley production (kg)*		*P*-value
		Only for home consumption	Only for sale	For home consumption and sale	For home consumption	For sale	
Misha	120	19.7	12.5	67.8	329	361	0.167
Gumer	123	34.1	22	43.9	816	1451	***
Hetosa	114	5.3	16.7	78.0	2057	2450	*
Total (3 districts)	357	20.5	16.8	62.7	1067	1420	***

*Mean amount of barley produced at household level for home consumption and for sale were compared by independent sample *t*-test. Value in level of significance are ***P < 0.001, *p < 0.05 and mean values with *P* > 0.05 are not significantly different and their respective p-value is shown

### End use: local foods and drinks prepared from varieties

Different barley varieties are used for various dishes and beverages. Farmers are aware with the barley varieties that they grow and how they are used. Table 6 lists the most widely recognized barley varieties, as well as the local meals and beverages made from them.

**Table 6:** Local foods and beverages prepared from most commonly cited barley

District	Barley variety	# record	End uses/local foods and drinks prepared barley
Misha district in Hadiya zone	*Awodo*	106	*Kolo—*roasted barley grain used as snacks *Beso—*meal prepared from flour of lightly roasted barley grain mixed with water *Kinche—*Ethiopian breakfast meal prepared from roasted and cracked barley boiled using either water or milk *Borde—*beverage prepared from traditional fermented barley *Chuko—*roasted barley flour (*Beso*) mixed with spiced butter to a stiff ball *Bread—*food made of flour, water and yeast mixed together and baked *Enjera –*thin Ethiopian bread *Genfo—*a thick porridge prepared by mixing fine flour of slightly roasted barley grain with boiling water and stirring until it smooth and thick *Anekalla—*roasted barley grain mixed with butter and used as snacks *Bullo—*flour boiled water
	*Du’uyya*	98	*Keneto—*non-alcoholic drink extracted from deeply roasted barley grain *Karebo*—a thin drink prepared from slightly fermented flour of roasted grain *Ayidara—*low alcoholic beverage made from malt barley *Borde*
	*Nazena*	78	*Tella—*fermented alcoholic beverage *Udurgufo—*large rounded bread that is baked on a flat surface in an oven *Karebo, Kolo, Borde*, *Bullo, Ayidara*
	*Mirt-zer*	69	*Shameta—*low alcoholic beverage made by overnight fermentation of roasted barley flour *Kinche, Kolo, Beso, Borde*
Gumer district in Gurage zone	*Jimua-Tikur*	87	*Kolo, Tella, Karebo, Shameta*
	*Shege*	81	*Kolo, Kinche*
	*Nech-Senef*	62	*Kolo*
	*Awodo*	56	*Shorba*—a kind of a hot soup made from coarsely grounded grain *Genfo*, *Kolo*, *Enjera*, *Chuko*, *Beso, Kinche*
Hetosa district in Arsi Zone	*Walia*	25	*Akayi* (*Kolo*), *Marka* (*Genfo*), *Bedena* (*Enjera*), *Bacho* (*Beso*), *Shaffe* (*Chuko*), *Shorba*
	*Kabe*	19	*Akayi* (*Kolo*), *Marka* (*Genfo*), *Kinche*, *Shorba*
	*Wolkari*	10	*Akayi* (*Kolo*), *Bacho* (*Beso*), *Marka* (*Genfo*), *Kinche*, *Keneto*, *Kure* (*Karebo*)
	*Eboni*	9	*Akayi (Kolo), Marka (Genfo), Bedena (Enjera), Beso, Kinche, Chuko*

### Seed selection and conservation practice of barley

**Fig. 4:**
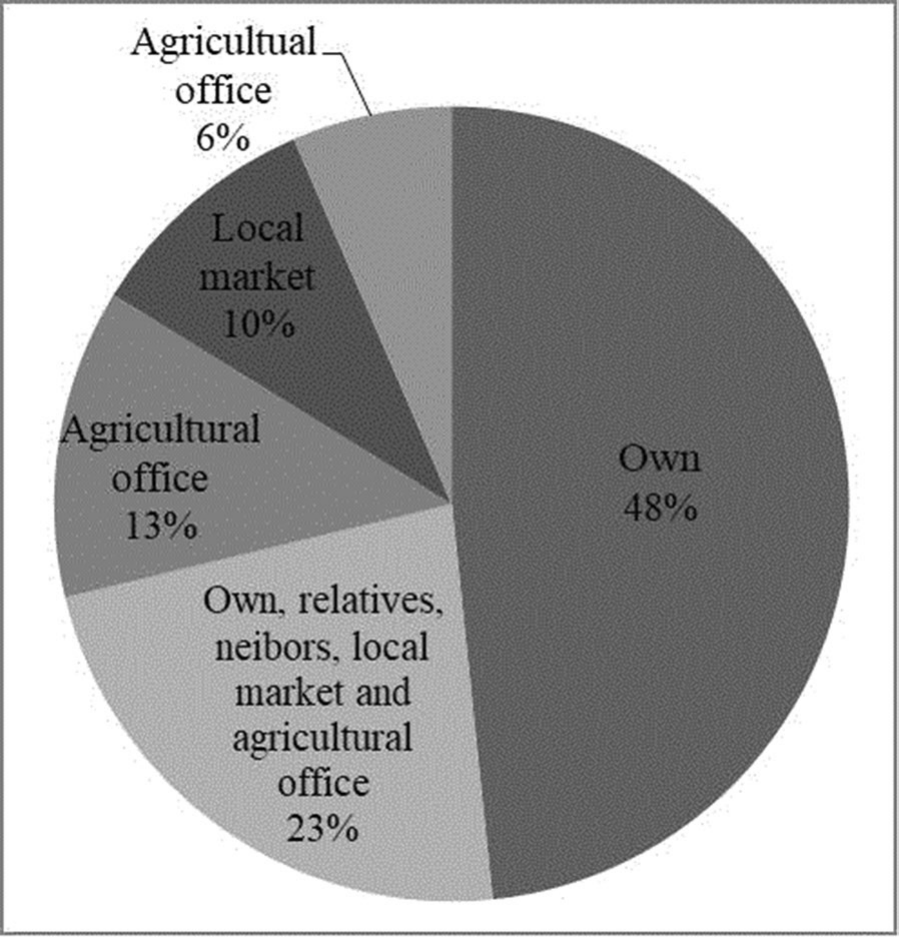
Barley seed sources for cultivation

The majority of farmers (48%) save their own barley seeds for the next growing season, unless farmers want to change the barley variety that the use. The selection was made after harvesting grain yield. Every year, 10% of the farmers buy barley seed for sowing from local markets. Only 6% of farmers receive better barley variety seeds from the agricultural office. Others cultivate their own local barley seeds as well as those obtained from markets, neighbors, and the district's agriculture sector each year (Fig. 4). Some of the farmers store their seed in “Shat”, traditionally made from bamboo and animal dung, while others stored it in plastic sacks for the next sowing season. To boost soil fertility and maximize productivity, farmers cultivate barley types in rotation with other crops. The seed is sawn after the farm has been plowed 3–5 times by oxen and hand hoe land preparation.

## Discussion

In this study, farmers’ knowledge of barley was recorded from the Misha district in the Hadiya zone, the Gumer district in the Gurage, and Hetosa in the Arsi. Farmer knowledge and a crop improvement program can be combined for sustainable and nutritious food supplies in the face of climate change. Bringing farmer knowledge to crop improvement and conservation increases the chances that new varieties will be adopted, making crop improvement more effective without compromising the conservation of existing genetic resources [30]. The proportion of male farmers who produce barley was significantly higher than that of female farmers (*x*^2^ = 91.77, *df* = 1, *p* < 0.001). The age group (41–60 years) has a considerably greater proportion of farmers who grow barley varieties (*x*^2^ = 28.38, *df* = 2, P < 0.001). The age of the farmers was directly correlated with the barley cultivation experience of farmers (*r* = 0.894, *P* < 0.001) but negatively correlated with the number of barley varieties (*r* = − 0.181, *P* < 0.001). More educated farmers have less experience in the cultivation of barley landraces (*r* = − 0.375, *p* < 0.001), indicating that older farmers have more experience in the cultivation of a greater number of barely varieties than the younger ones. This indicates that the majority of the barley growers are adults. A study conducted in the Bale high lands, Ethiopia also indicated that most barley cultivating farmers (88.8%) were in the adult age group (above 40 years) [24]. This study's result showed that the majority of farmers (91.3%) who cultivate barely were married. A similar study in Welmera and Ejjera districts also showed that the majority (78%) of farmers who used to grow barley were married [22]. This may imply that most local farmers who cultivate barley are married. A larger majority of the farmers (48.5%) followed primary education along with agricultural work. This could have aided them in carrying out knowledge-based agricultural work passed down from their parents.

The majority of farmers grow barley on 0.5–0.75 hectares, followed by those who grow on > 0.75 hectares. Farmers who produce barley on more than one hectare have been found only in the Hetosa district. None of the farmers in this district grow barley on less than 0.5 hectares of land. In line with this, previously it was reported that Arsi was among those areas of Ethiopia that produced a larger amount of barley [31]. The greatest number of farmers (59.7%) in the Hetosa district, in particular, reported having been growing barley for 31 years or more, revealing their experience in barley cultivation and thus providing useful information regarding barley varieties grown over 20–30 years ago. The crop is produced in all regions of Ethiopia, covering 1,018,752.94 hectares of land with 1,781,652.208 tons of annual production [32].

Farmers designate local names based on characteristics of barley such as spikelet length, dehulling, digestibility, row type, seed size, seed color, yield, kernel color, plant height, and place of origin, use-values, and the person who first introduced the barley to the area. According to the results of various studies, Ethiopian farmers utilize the majority of these traits to identify, name, and describe barley varieties in their areas [7, 9, 21, 22, 33]. Similar research, which was done on eset [34] and on beans [35], reported that farmers attach local names with different characteristics that are used to describe and differentiate. According to farmers, knowing such characteristics has practical importance for the cultivation of various varieties. For example, knowing the maturation time is critical for minimizing harvest loss.

During this study, 68 barley varieties (landraces and improved) with distinct local names were identified and described by farmers (Table 2). The number of barley varieties identified in each district (Misha district: 22, Gumer district: 15, and Hetosa district: 31) was comparable to or greater than the number of barley varieties previously reported from the northwestern (24) [7], West Shewa [15], Bale (25) [23], and northeastern (15) [36] highlands of Ethiopia.

Most farmers in the Misha and the Gumer districts grow more barley at the household level on lower farmland sizes (Fig. 3 and Table 3) than in the Hetosa district. The reason could be that these districts mostly produce barley through crop-livestock mixed farming systems [37]. In the Hetosa district, the majority of farmers cultivate only high-yielding improved commercial barley varieties on larger farms. Farmers in Uganda primarily grow improved cassava varieties that are known to be high-yielding, and they do not care much about selection, on-farm retention, or conservation because improved varieties are distributed by government and non-government organizations on a regular basis [38]. In major wheat-growing parts of Ethiopia, 72% of farmers grew only one commercial wheat cultivar [39]. This suggests that while distributing improved commercial cultivars, special attention should be paid to the conservation of existing genetic resources.

The mean number of barley varieties (2.66) currently growing at the household level is significantly lower than the mean number of barley varieties (4.77) cultivated at the household level before 2–3 decades (p 0.001). This indicates that the number of barley varieties has been decreasing over time. A similar case was reported from the Gamo highlands, southern Ethiopia [33], where the average number of barley varieties on-farm declined to 2.3. In Northern Gondor, 85% of farmers reported that the number of barley landraces was declining in their locality [40]. According to farmers, the introduction of improved commercial barley cultivars was the primary cause of the decline in the number of local barley types. Similar studies on barley in different parts of Ethiopia [9, 23, 41, 42] reported a reduction in the number of barley local varieties due to displacement by the introduction of improved barley varieties, indicating that the introduction of improved varieties to localities is becoming the main cause of genetic erosion of barley local varieties.

The decline of native barley varieties is attributed to several interrelated factors, such as the widespread introduction of improved and exotic varieties; recent climate change that has led to habitat destruction and periodic drought; and advances in agricultural technology, including the shift to the use of mechanized farming [23]. Another reason for the destruction of local varieties of barley was the decrease in soil fertility, which was mentioned by 24.2% of farmers. As the loss of soil fertility occasionally results in reduced yields of local varieties of barley, farmers focus on improved varieties. This reason has also been previously reported by various authors studying local barley cultivars in different parts of Ethiopia [15, 40].

The results of the current study showed that 62.7% of farmers grow barley for both home consumption and sale. The barley production data from the study districts, however, showed that the amount of barley produced for sale (1420 kg) was significantly greater than that produced for home consumption (1067 kg) (*p* 0.001). It was observed that farmers with small land sizes grow barley for home consumption. A similar observation was made in mid-western Uganda in that farmers with a small acreage of land grow cassava mostly for home consumption [38].

Farmers know not only the cultivation of barley but also the end-use of the barley that they grow. They prepare various well-appreciated local foods and drinks from barley varieties that they grow. In a similar study conducted in two districts of West Shewa, no other cereal crop can be processed into so many different forms of food so as barley [15]. Various localized Ethiopian traditional foodstuff and local drinks, which have been prepared from barley varieties, were reported from different parts of Ethiopia [21, 22, 40]. Farmers use their own knowledge that was gained through experiences for seed selection and conservation. All of them keep their own barley to the next growing season, selecting after the harvesting of the whole yield. Only 6% of farmers obtain barley seed from the agricultural office. Others use their own barley seed as well as from market, neighbors and agriculture sector of the districts (Fig. 4). Barley that the farmers grow is used to make various types of home-made foods and local drinks. Farmers are aware the barley varieties that they cultivate and use is served as a solution for protecting food security, meeting future food needs and providing social benefits (Table 6). Keeping a few but a variety of plants for food security and growing local varieties mostly for home consumption have ensured the conservation of local varieties [21].

## Conclusion

Barley cultivation practices depend on and are acquired along with gender and age groups as revealed by the significantly higher percentage of male farmers cultivating barley in adult age. Farmers have accumulated experience from farming barley for the past 20–30 years or more. Older farmers have more experience in cultivation of more number of barely varieties than the younger ones. More educated farmers were younger ones who grow the improved barley varieties on larger farm land size and they have less experience in cultivation of barley landraces. Farmers use different characteristics of barley to assign local names and attach the names with a practical implication for the cultivation of barley varieties. They identified and described 68 barley varieties with various local names. Although various localized but well appreciated, homemade local foods and drinks were prepared from seed grains of barley, the majority of farmers give attention to only commercial barley cultivars. Thus, an introduction of improved barley cultivars has been declining the number of barley local varieties, leading to genetic erosion of barley local varieties although it has economic importance. Farmers have their own knowledge that was gained through experiences in seed selection and conservation. Farmers' knowledge might thus be viewed as an opportunity for barley genetic resource conservation through a participatory strategy to expand barley farming and improve local barley varieties for future use.

## Data Availability

All data collected for this study were analyzed, interpreted, and included in this manuscript, but other datasets used and/or analyzed during the current study are available from the corresponding author on reasonable request.
